# Sex-Specific Association of Alcohol Use Disorder With Suicide Mortality: A Systematic Review and Meta-Analysis

**DOI:** 10.1001/jamanetworkopen.2024.1941

**Published:** 2024-03-12

**Authors:** Shannon Lange, Kawon V. Kim, Aurélie M. Lasserre, Heather Orpana, Courtney Bagge, Michael Roerecke, Jürgen Rehm

**Affiliations:** 1 Institute for Mental Health Policy Research, Centre for Addiction and Mental Health, Toronto, Ontario, Canada; 2 Campbell Family Mental Health Research Institute, Centre for Addiction and Mental Health, Toronto, Ontario, Canada; 3 Department of Psychiatry, University of Toronto, Toronto, Ontario, Canada; 4 Institute of Medical Science, University of Toronto, Toronto, Ontario, Canada; 5 Addiction Medicine, Department of Psychiatry, Lausanne University Hospital, Lausanne, Switzerland; 6 Public Health Agency of Canada, Ottawa, Ontario, Canada; 7 School of Psychology, University of Ottawa, Ottawa, Ontario, Canada; 8 Department of Psychiatry, University of Michigan Medical School, Ann Arbor; 9 Center for Clinical Management Research, Department of Veterans Affairs, Ann Arbor, Michigan; 10 Dalla Lana School of Public Health, University of Toronto, Toronto, Ontario, Canada; 11 Program on Substance Abuse and World Health Organization European Region Collaboration Centre, Public Health Agency of Catalonia, Barcelona, Spain; 12 Zentrum für Interdisziplinäre Suchtforschung, Universitätsklinikum Hamburg-Eppendorf, Hamburg, Germany

## Abstract

**Importance:**

Despite individual studies suggesting that sex differences exist in the association between alcohol use disorder (AUD) and suicide, most existing systematic reviews and meta-analyses have reported associations across the sexes.

**Objective:**

To estimate the sex-specific association between AUD and suicide mortality.

**Data Sources:**

Embase, MEDLINE (including MEDLINE In-Process), PsycINFO, PubMed, and Web of Science were searched from database inception to April 27, 2022.

**Study Selection:**

Inclusion criteria consisted of the following: (1) original, quantitative study, (2) inclusion of a measure of association and its corresponding measure of variability (or sufficient data to calculate these [eg, 95% CI]), and (3) results stratified by sex.

**Data Extraction and Synthesis:**

Data extraction was completed by one reviewer and then cross-checked by a second reviewer. Risk of bias was assessed by study design. Categorical random-effects meta-analyses were conducted to obtain sex-specific pooled estimates of the association between AUD and suicide mortality risk. Methodological moderators (ie, study design and comparator group) were assessed using sex-stratified meta-regressions.

**Main Outcomes and Measures:**

The association between AUD and suicide mortality.

**Results:**

A total of 16 347 unique records were identified in the systematic search; 24 studies were ultimately included for 37 870 699 participants (59.7% male and 40.3% female) (23 risk estimates for male and 17 for female participants). Participants ranged in age from 15 years to 65 years or older. Sex-specific meta-regression models indicated that study design (ie, longitudinal vs cross-sectional study design) affected the observed association between AUD and suicide mortality for both male participants (log odds ratio, 0.68 [95% CI, 0.08-1.28]; *P* = .03) and female participants (log odds ratio, 1.41 [95% CI, 0.57-2.24]; *P* < .001). For males and females, among longitudinal studies, the pooled odds ratios were 2.68 (95% CI, 1.86-3.87; *I*^2^ = 99% [n = 14]) and 2.39 (95% CI, 1.50-3.81; *I*^2^ = 90% [n = 11]), respectively.

**Conclusions and Relevance:**

This systematic review and meta-analysis yielded substantive evidence that AUD was associated with suicide mortality and that the association was similar across the sexes. The findings underscore the importance of identifying and treating AUD as part of a comprehensive suicide prevention strategy.

## Key Points

**Question:**

Does alcohol use disorder (AUD) have a sex-specific association with suicide mortality?

**Findings:**

This systematic review and meta-analysis found that sex differences in the association of AUD with suicide mortality were a function of bias introduced by the study design. Among longitudinal studies, both sexes with AUD had statistically significantly higher odds of dying by suicide compared with their counterparts without AUD.

**Meaning:**

Alcohol use disorder is associated with similar heightened odds of suicide mortality for males and females, underscoring the need for simple routine screening measures that could have an important impact in terms of lives saved.

## Teaser

This systemic review and meta-analysis estimates the sex-specific association between alcohol use disorder and suicide mortality.

## Introduction

According to the World Health Organization, more than 700 000 people died by suicide globally in 2019, with an age-standardized suicide mortality rate of 9.0 per 100 000 population (12.6 per 100 000 males and 5.4 per 100 000 females).^1^ Suicide is the only mental health indicator of the United Nations Sustainable Development Goals (Target 3.4: “By 2030, reduce premature mortality from noncommunicable diseases by one-third through prevention and treatment and promote mental health and well-being”).^2^ In the Sustainable Development Goals, suicide functions as an indicator for mental disorders, underscoring the importance of understanding its association with specific psychiatric conditions—for instance, alcohol use disorder (AUD). Alcohol use disorder has been identified as an important risk factor for suicide.^3,4,5^ A meta-analysis^5^ published in 2015 found a significant association between AUD and death by suicide, with a 2.6-fold (95% CI, 2.0-3.2) higher risk among individuals with an AUD, compared with those without an AUD.

However, existing studies suggest that the association may be stronger for female individuals.^6,7,8^ For instance, in a meta-analysis looking at cause-specific mortality risk among individuals in treatment for AUD, Roerecke and Rehm^8^ found that the standardized mortality ratio of death by suicide associated with AUD was about 2 times higher for female individuals than for male individuals. The heightened risk of suicide among female individuals with AUD compared with the risk of suicide among male individuals with AUD, relative to those without AUD, could be due to the fact that female individuals have a heightened susceptibility to the effects of alcohol,^9,10^ evident by a more rapid progression of AUD^11^ and an earlier onset of alcohol-related health and psychosocial complications.^12^ Additionally, for some countries it has been suggested that women with an AUD are less likely to access AUD treatment services compared with their male counterparts,^13,14^ which could be due to women with an AUD being more likely to experience social stigmatization and weakened social integration than men.^15,16^

Although previous meta-analyses have found that AUD is a risk factor for suicide,^3,5,7,8,17,18,19^ the majority have pooled risk estimates across both sexes. Ignoring sex-related differences in the association between AUD and suicide may introduce bias in risk estimates and limit the applicability of the evidence for clinical or public health practice.^20^ The few studies that have stratified their meta-analyses by sex are either outdated^7,8^ or failed to include relevant studies fitting their exposure definition.^19^

Accordingly, the purpose of the present study was to estimate the sex-specific association between AUD and suicide mortality. The following hypotheses were tested: (1) individuals with AUD would have higher odds of dying by suicide vs those without and (2) the association between AUD and suicide mortality would be higher for females than for males. The present study is part of a larger systematic review investigating various measures of alcohol consumption and their association with the risk of death by suicide.^21^

## Methods

This systematic review followed the Preferred Reporting Items for Systematic Review and Meta-Analysis (PRISMA) 2022 statement. The protocol has been published elsewhere^21^ and was registered with the International Prospective Register of Systematic Reviews (CRD42022320918).

### Search Strategy

A systematic literature search was performed in Embase, MEDLINE (including MEDLINE In-Process), PsycINFO, PubMed, and Web of Science from database inception to April 27, 2022. The search strategies incorporated a combination of medical subject headings and keywords for *alcohol use* and *suicide* (eMethods in Supplement 1). Manual reviews of citations in the articles deemed relevant and the studies included in related reviews and meta-analyses were conducted. In addition to searching for published data, we searched for unpublished data by contacting subject matter experts and investigators and screening conference proceedings. The search results were imported into EndNote 20.3 for removal of deduplicates.^22^

### Study Selection

Two individuals (K.V.K. and A.M.L.) were trained to screen titles and abstracts using batches of 100 randomly selected records. Training involved independent review and discussion of all discrepancies, until high agreement (eg, κ > 0.8)^23^ was reached. The titles and abstracts of the remaining records were then independently screened by a single reviewer. The same process was used for full-text screening. In cases of uncertainty, a discussion was had between both reviewers; third-party adjudication was used if an agreement could not be reached. Title and abstract screening was completed using EndNote 20.3,^22^ and full-text screening was performed in Covidence.^24^ Data extraction was completed by one investigator (K.V.K.) using a template created in Excel, version 2019 (Microsoft Corporation)^25^ (the template was initially piloted using 10 randomly selected studies) and cross-checked by a second investigator (A.M.L.). If there was inadequate reporting of data, data were unavailable (eg, effect estimate values were not published), or sex-specific estimates were not reported, corresponding authors were contacted.

### Eligibility Criteria

Our basic inclusion criteria consisted of (1) original, quantitative study; (2) inclusion of a measure of association and its corresponding measure of variability (or sufficient data to calculate these [eg, 95% CIs]); and (3) results stratified by sex. There were no restrictions on setting, language of publication, geographical location, or year of publication. Table 1 provides specific inclusion and exclusion criteria.

**Table 1.** Population, Interventions, Comparators, and Outcomes Criteria for Study Selection

Criteria	Inclusion criteria	Exclusion criteria
Population	Individuals ≥15 y of age and those whose cause of death was something other than suicide	None
Intervention or exposure	Lifetime AUD, including alcohol abuse and alcohol dependence	None
Comparators	Individuals without AUD in their lifetime	Individuals diagnosed or hospitalized with a SUD, other than AUD
Outcome	Death by suicide and sex-specific estimates	Death by suicide and suicide attempt cannot be disaggregated or individuals have an undetermined cause of death
Study design	Quantitative observational study designs: cohort, case-control, or cross-sectional	None
Other	Any language, any geographical region, and any year of publication	Overlapping sample with another study that has a more comprehensive sample or is more recent, or dissertation or conference abstract

Abbreviations: AUD, alcohol use disorder; SUD, substance use disorder.

#### Definitions

Acceptable definitions of AUD included the *Diagnostic and Statistical Manual of Mental Disorders* (Third and Fourth Editions) (*DSM*) (alcohol abuse and alcohol dependence), and *DSM-5* (AUD); *International Classification of Diseases, Ninth Revision* (*ICD-9*),^26^ (codes 303.0, 305.0, and 303.9), *ICD-10* (codes F10.0, F10.1, and F10.2),^27^ or *ICD-11* (codes 6C40.1 and 6C40.2)^28^ (harmful use of alcohol and alcohol dependence syndrome); medical opinion and accepted diagnostic tools as outlined in *Assessing Alcohol Problems: A Guide for Clinicians and Researchers*^29^; and people who seek or receive AUD treatment. Death by suicide was defined as death caused by any self-inflicted injurious behavior that was intended to kill oneself^30^ or applicable *ICD-9* (E950-E959),^26^ *ICD-10* (X60-X84 and Y87.0),^27^ or *ICD-11* (PB80-PD3Z)^28^ codes.

### Statistical Analysis

Sex-specific pooled odds ratio (OR) estimates were calculated by performing categorical random-effects meta-analyses. Hazard ratios, relative risks, and ORs were treated as equivalent measures of association. In the case where a study did not present estimates for AUD overall, but rather separate estimates for alcohol abuse and alcohol dependence, we first pooled them in a fixed-effects meta-analysis and included the resulting estimate in the main meta-analysis. Between-study heterogeneity was assessed using the *I*^2^ statistic, and publication bias was evaluated using the Egger test and a funnel plot to assess asymmetry. Sex-stratified random-effects meta-regression analyses were performed to assess the effect of study design and comparator group (ie, no lifetime AUD vs no AUD as assessed previously or at study baseline). If the meta-regression results demonstrated significant effects of these covariates, stratifying the meta-analysis accordingly was considered to reduce potential sources of heterogeneity. To test whether the OR was significantly higher for females than for males, the Wald test was used. Last, given that the conceptualization of suicide and in particular AUD has changed over the years with the iterations of the *DSM* and *ICD*, studies were ordered by study year in the forest plot to visually explore whether their association has changed over time. All analyses were performed using RStudio, version 1.3.1073 (R Project for Statistical Computing),^31^ and statistical significance was set at α = .05 (2-tailed).

### Risk of Bias

Risk of bias was independently assessed by 2 reviewers (K.V.K. and A.M.L.) using the risk of bias in nonrandomized studies of exposure (ROBINS-E) tool for cohort studies^32^ and the Joanna Briggs Institute critical appraisal tools for case-control and cross-sectional studies.^33^ The E-value method was used to estimate the severity of bias (particularly the bias due to potential confounding) that would be required, hypothetically, to shift the pooled estimate to the null.

## Results

### Study Selection and Characteristics

**Figure 1.**
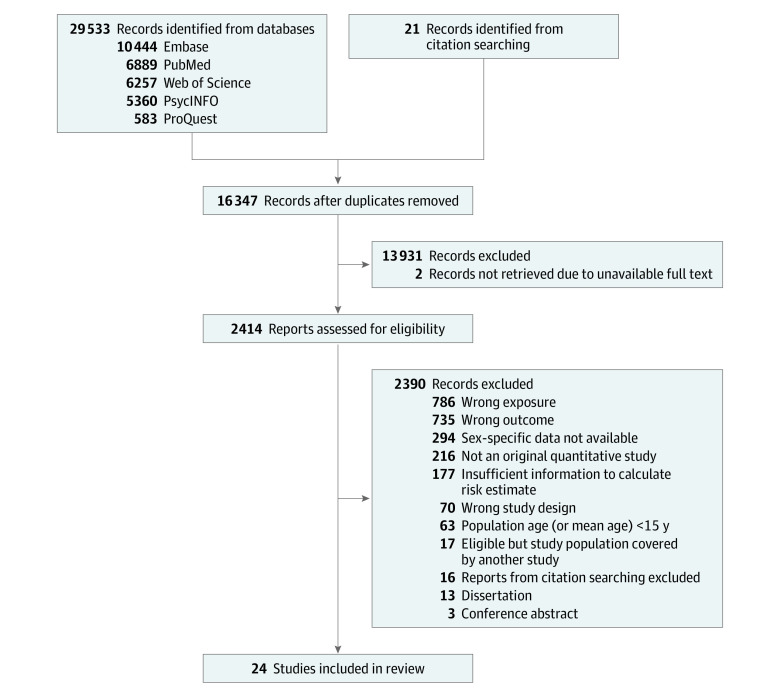
Study Flowchart

Our search strategy initially yielded 29 533 records; after removal of duplicates, 16 347 remained (Figure 1). After abstract screening and full-text review, 19 studies^34,35,36,37,38,39,40,41,42,43,44,45,46,47,48,49,50,51,52^ identified from the search strategy and 5 studies^53,54,55,56,57^ identified from manual reviews were included for analysis, for a total of 24 studies with 37 870 699 participants (59.7% male and 40.3% female) (Table 2). Participants ranged in age from 15 years to 65 years or older. There were 11 cohort studies,^35,36,39,41,43,45,47,52,53,54,55^ 4 longitudinal case-control studies,^34,40,46,57^ 8 cross-sectional case-control studies,^37,38,42,44,48,49,50,56^ and 1 cross-sectional prevalence study.^51^ In 10 studies,^38,42,44,45,47,49,52,53,56,57^ individuals with an AUD diagnosis were compared with individuals without an AUD as determined at study baseline or during a specified retrospective period of time (eg, last 12 months). In the remaining 14 studies,^34,35,36,37,39,40,41,43,46,48,50,51,54,55^ the comparator group consisted of individuals without lifetime AUD (ie, unspecified period of time). While all studies investigated death by suicide as the outcome, 6 studies^34,36,39,41,43,50^ combined suicide deaths and undetermined cause of death, and in 6 additional studies,^37,38,40,46,56,57^ it could not be clearly determined whether the outcome consisted only of suicide deaths or included undetermined cause of death as well. Cases of death by suicide were compared with matched living controls in 8 studies,^40,42,44,46,48,49,56,57^ living individuals or those with other causes of death in 12 studies,^34,35,36,39,41,43,45,47,50,52,54,55^ those with natural causes of death in 1 study,^51^ and finally those without a suicide attempt in the past 3 months.^53^

**Table 2.** Characteristics of All Included Studies

Source	Country	Study design	Population	Age category^a^	Study years		No. of participants	Case definition	Control definition	Unexposed group	Covariates
					Start	End					
Allebeck et al,^34^ 1987	Sweden	Longitudinal case-control	Patients	All ages	1971	1981	Male: 46; female: 50	Suicide plus undetermined deaths	Living or death by all other COD	No AUD (lifetime)	None
Bohnert et al,^35^ 2017	US	Cohort	Veterans	All ages	2006	2011	Male: 4 460 447; female: 402 639	Suicide	Living or death by all other COD	No AUD (lifetime)	Age; Charlson Comorbidity Index; presence or absence of depression, schizophrenia, bipolar disorder, PTSD, or other anxiety disorders
Chen et al,^53^ 2020	Taiwan	Cohort	Patients	All ages	2012	2018	Female: 2780	Suicide	No suicide attempt, past 3 y	No AUD (previous or assessed at baseline)	Prior methadone treatment; history of depressive disorders or treated suicide attempts; retaining in methadone treatment; age, educational level, marital status, employment, income, and having a child younger than 12 y
Crump et al,^36^ 2021	Sweden	Cohort	General	All ages	2003	2016	Male: 3 403 918; female: 3 543 273	Suicide plus undetermined deaths	Living or death by all other COD	No AUD (lifetime)	Age, marital status, educational level, employment, and income; psychiatric, SUD, or somatic comorbidities
Dalca et al,^37^ 2013	Canada	Cross-sectional case-control	Patients	Middle-aged	NS^b^	NA	Male: 248; female: 80	NS^b^	Matched living control	No AUD (lifetime)	None
Dumais et al,^38^ 2005	Canada	Cross-sectional case-control	Patients	All ages	2000-2004	NA	Male: 178	NS^b^	Matched living control	No AUD (previous or assessed at baseline)	Age; aggressive behavior; impulsivity; cluster B personality disorder; drug abuse or dependence in last 6 mo
Edwards et al,^39^ 2020	Sweden	Cohort	General	Young	1965-1985	2012	Male: 1 145 634; female: 1 084 246	Suicide plus undetermined deaths	Living or death by all other COD	No AUD (lifetime)	Drug use, affective, psychotic, personality phobia and anxiety, or other psychiatric disorders; birth year; mean parental educational level
Feodor Nilsson et al,^54^ 2014	Denmark	Cohort	Homeless	All ages	1999	2008	Male: 22 508; female: 9439	Suicide	Living or death by all other COD	No AUD (lifetime)	Age at first homeless shelter contact recorded in the Danish Homeless Register; country of origin and main source of income; history of abuse of all other types of drugs; schizophrenia spectrum, affective, or personality disorder
Heu et al,^40^ 2018	Sweden	Longitudinal case-control	Patients	Middle-aged	1956	2010	Male: 88; female: 112	NS^b^	Matched living control	No AUD (lifetime)	None
Holmstrand et al,^41^ 2015	Sweden	Cohort	General	All ages	1947 and 1957	2011	Male: 1823; female: 1740	Suicide plus undetermined deaths	Living or death by all other COD	No AUD (lifetime)	Comorbid depression and psychosis
Ilgen et al,^55^ 2010	US	Cohort	Veterans	All ages	2000	2006	Male: 2 962 810; female: 329 081	Suicide	Living or death by all other COD	No AUD (lifetime)	Age group
Kim et al,^56^ 2003	Canada	Cross-sectional case-control	General	All ages	1989	NA	Male: 197	NS^b^	Matched living control	No AUD (previous or assessed at baseline)	None
Kõlves et al,^42^ 2006	Estonia	Cross-sectional case-control	General	All ages	1999 and 2002-2003^c^	NA	Male: 668; female: 139	Suicide	Matched living control	No AUD (previous or assessed at baseline)	Marital and employment status
Lannoy et al,^43^ 2022	Sweden	Cohort	General	All ages	1979	2015	Male: 903 333	Suicide plus undetermined deaths	Living or death by all other COD	No AUD (lifetime)	Year of birth; mean parental educational level; resilience
Lynch et al,^44^ 2020	US	Cross-sectional case-control	General	All ages	2000-2013	NA	Male: 129 052; female: 141 020	Suicide	Matched living control	No AUD (previous or assessed at baseline)	Age, educational level, and poverty level; Charlson Comorbidity Index; psychiatric diagnoses
Mukamal et al,^45^ 2007	US	Cohort	Health care workers	All ages	1986	2002	Male: 47 654	Suicide	Living or death by all other COD	No AUD (previous or assessed at baseline)	None
Park et al,^46^ 2008	South Korea	Longitudinal case-control	General	All ages	2002-2003	2003-2004	Male: 86 933; female: 39 820	NS^b^	Matched living control	No AUD (lifetime)	Residence area and economic status; psychiatric disease (bipolar disorder, schizophrenia, alcohol abuse); cancer
Penttinen et al,^57^ 2006	Finland	Longitudinal case-control	Patients	Young	1979-1980	1992	Male: 100	NS^b^	Matched living control	No AUD (previous or assessed at baseline)	Date of birth (±3 y); smoking habit; social status; county of residence
Phillips et al,^47^ 2017	US	Cohort	Military	Young	2001-2010	2001-2012	Male: 108 930	Suicide	Living or death by all other COD	No AUD (previous or assessed at baseline)	None
Schneider et al,^48^ 2005	Germany	Cross-sectional case-control	General	All ages	1999-2000	NA	Male: 300; female: 202	Suicide	Matched living control	No AUD (lifetime)	Age and living area
Shaffer et al,^49^ 1996	US	Cross-sectional case-control	General	Young	1984-1986^d^	NA	Male: 196	Suicide	Matched living control	No AUD (previous or assessed at baseline)	Socioeconomic status; ethnicity; age; previous suicide attempt, runaway behavior, or recklessness; computer-generated parent-informed diagnostic disorder groupings (mood, disruptive, substance abuse, and anxiety)
Waern et al,^50^ 2003	Sweden	Cross-sectional case-control	General	Older	1994-1996	NA	Male: 130; female: 108	Suicide plus undetermined deaths	Living or death by all other COD	No AUD (lifetime)	Age; major depression; family discord; operationally defined serious physical illness
Yoon et al,^51^ 2011	US	Cross-sectional prevalence	Deceased	All ages	1999-2006	NA	Male: 9 282 404; female: 9 682 384	Suicide	Death by natural cause	No AUD (lifetime)	None
Zaheer et al,^52^ 2020	Canada	Cohort	Patients	All ages	1993	2012	Male: 45 633; female: 30 356	Suicide	Living or death by all other COD	No AUD (previous or assessed at baseline)	Age at diagnosis; income quintile; rural vs urban; ADG score; drug use, mood, or personality disorder; any outpatient psychiatrist contact; any mental health hospitalization; any suicidal behavior requiring ED or hospital care

Abbreviations: ADG, Aggregated Diagnosis Group; AUD, alcohol use disorder; COD, cause of death; ED, emergency department; NA, not applicable; NS, not specified; PTSD, posttraumatic stress disorder; SUD, substance use disorder.

^a^ Young indicates 15 to 34 years of age; middle-aged, 35 to 64 years of age; older, 65 years or older; and all ages, 2 or more age groups.

^b^ Indicates that it could not be clearly determined whether the outcome was comprised of only suicide deaths or included undetermined cause of death as well.

^c^ Indicates 1999 for cases and 2002 to 2003 for controls.

^d^ Indicates June 1, 1984, to May 31, 1986.

### Participants’ Characteristics

Most studies included participants from the general population or from a nationally representative sample, followed by studies of a patient population,^34,37,38,40,52,53,57^ active^47^ or veteran^35,55^ military personnel, individuals experiencing homelessness,^54^ deceased individuals,^51^ and health professionals.^45^ The age range of the individuals in the studies varied, with 4 studies^39,47,49,57^ limited to adolescents and young adults (ie, aged 15-34 years), 2 studies^37,40^ limited to middle-aged adults (ie, aged 35-64 years), 1 study^50^ limited to older adults (ie, 65 years or older), and the remaining majority of studies including participants from 2 or 3 of the aforementioned age groups. Sex-specific estimates for both sexes were reported by 16 studies,^34,35,36,37,39,40,41,42,44,46,48,50,51,52,54,55^ while 7 studies^38,43,45,47,49,56,57^ reported estimates for males only, and 1 study^53^ reported estimates for females only. In total, 23 estimates were extracted for males and 17 for females.

### Risk of Bias

Overall, potential confounding was identified as the source of potential bias in the included studies; this was the case for 4 cohort studies^41,45,47,55^ (eFigure 1 in Supplement 1), 6 case-control studies^34,37,40,46,56,57^ (eTable 1 in Supplement 1), and 1 cross-sectional prevalence study^51^ (eTable 2 in Supplement 1). To account for the estimated ORs from longitudinal studies for males and females, an unmeasured confounder that was associated with both AUD and suicide by an OR of 4.8-fold and 4.2-fold, respectively, above and beyond the measured confounders, would be required to shift the pooled estimate to the null, but weaker confounding would not do so (eTable 3 in Supplement 1). In addition, 1 case-control study^37^ used different criteria for selecting cases and controls.

### Sex-Specific Association Between AUD and Suicide Mortality

**Figure 2.**
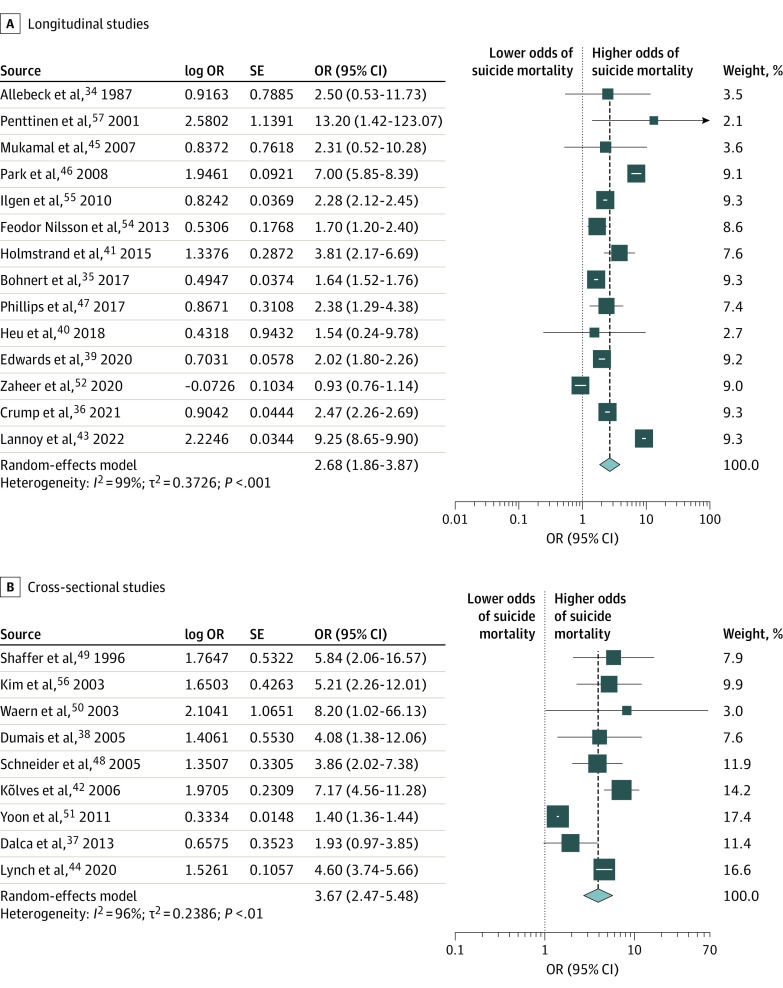
Association of Alcohol Use Disorder With Suicide Mortality Risk Among Male Individuals by Study Design OR indicates odds ratio. Error bars indicate 95% CI.

**Figure 3.**
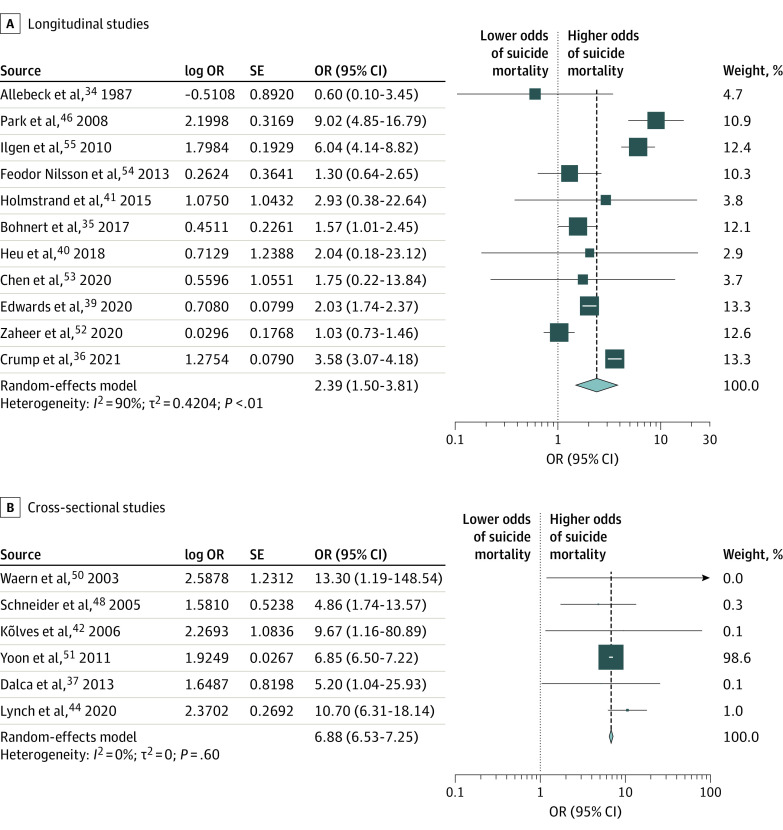
Association of Alcohol Use Disorder With Suicide Mortality Risk Among Female Individuals by Study Design OR indicates odds ratio. Error bars indicate 95% CI.

The pooled OR from the random-effects model meta-analysis was 3.03 (95% CI, 2.31-3.99; *I*^2^ = 99%) for male individuals and 3.41 (95% CI, 2.25-5.15; I^2^ = 96%) for female individuals (eFigures 2 and 3 in Supplement 1). Sex-specific Egger tests (male individuals: *t* = 1.31 [*P* = .20]; female individuals: *t* = −1.84 [*P* = .10]) and funnel plots indicated that there was no evidence of publication bias (eFigures 4 and 5 in Supplement 1). Sex-specific meta-regression models including study design type and nonexposed group type revealed a significant effect of study design type (ie, longitudinal vs cross-sectional) for both male individuals (log OR, 0.68 [95% CI, 0.08-1.28]; *P* = .03) and female individuals (log OR, 1.41 [95% CI, 0.57-2.24]; *P* < .001), and nonsignificant effect of nonexposed group type for both sexes (eTable 4 in Supplement 1). Accordingly, the final models were stratified by study design (ie, longitudinal study design [cohort and longitudinal case-control study designs] and cross-sectional study design [cross-sectional case-control and prevalence study designs]) for both sexes. For male individuals, the pooled OR among longitudinal studies was 2.68 (95% CI, 1.86-3.87; *I*^2^ = 99%) and among cross-sectional studies was 3.67 (95% CI, 2.47-5.48; *I*^2^ = 96%) (Figure 2). Among female individuals, the pooled OR among longitudinal studies was 2.39 (95% CI, 1.50-3.81; *I*^2^ = 90%) and among cross-sectional studies was 6.88 (95% CI, 6.53-7.25; *I*^2^ = 0%) (Figure 3). As per the forest plots, the association between AUD and suicide mortality does not appear to have changed over time, suggesting that the changes in their conceptualization over time did not bias the current findings.

We found that the pooled OR estimates from longitudinal studies for male and female individuals were not statistically significantly different from one another (difference [OR], 0.29 [95% CI, −0.92 to 1.50]; *P* = .64). However, in contrast, the pooled OR estimates from cross-sectional studies for male and female individuals were statistically significantly different from one another (difference [OR], −3.21 [95% CI, −4.46 to −1.96]; *P* < .001), with female individuals having a higher OR.

## Discussion

Based on pooled risk estimates derived from studies using a longitudinal design, AUD is associated with greater than a 2-fold higher odds of death by suicide for both male and female individuals, with no statistical difference between the sexes. Sex differences were apparent in the pooled estimates derived from cross-sectional studies, with female individuals with an AUD having significantly higher odds of dying by suicide compared with their male counterparts. However, causality cannot be inferred with such a study design, as temporality cannot be determined. Therefore, while a sex difference in the association between AUD and suicide mortality was hypothesized based on some previous studies, it appears that any such differences are a function of bias introduced by the study design, and do not reflect causal impact.

Regardless of there not being a statistically significant difference between male and female individuals in the association between AUD and suicide mortality, it is clear that individuals with AUD have elevated odds of dying by suicide compared with those without an AUD. Coupled with the finding that over 80% of individuals who die by suicide had contact with the health care system in the year prior to their suicide,^58,59^ there are clear implications for suicide prevention efforts within the health care system. For instance, the temporal relationship that can be discerned from the meta-analyses of longitudinal studies speaks to the importance of a risk management approach among individuals with an AUD. However, suicide risk management is only possible if AUD is identified. Thus, it is imperative to screen for alcohol use among individuals presenting with depression and/or suicidal ideation. Having said that, the inverse is also true, and depression and/or suicidal ideation should also be screened among individuals with AUD. These rather simple screening measures could have an important impact in terms of lives saved.

It is important to point out that the current study represents the association between one of the predisposing dimensions of alcohol use and suicide mortality. Although not necessarily distinct, the association between average daily amount of alcohol consumed per day and suicide and the shape of the dose-response curve remain largely unknown. Moreover, it is not clear whether the association between AUD and suicide mortality risk is due to the physiological effects of alcohol, the adverse social, occupational, or health consequences that go along with an AUD diagnosis, or both. It is possible that the lack of difference between the sexes in the focal relationship under investigation when longitudinal study designs are considered alone is that the long-term consequences of AUD (ie, the adverse social, occupational, or health consequences) are equally detrimental to males and females. It is also possible that the cross-sectional studies are capturing more of the physiological effects of alcohol, which are known to differ by sex, and this is reflected in the sex differences observed when cross-sectional study designs are considered alone.

In the present study, the term *sex* has been used throughout. This was used as the outcome of interest is a mortality outcome, typically ascertained via official statistics or death report data. With such data, biological sex is discerned and documented. However, the role of gender and more specifically gender-related factors (eg, social norms surrounding both alcohol use and suicide) should not be overlooked, nor should the terms *sex* and *gender* be conflated (as is a possibility in some on the studies included herein). Further, research suggests that members of sexual minority groups experience a higher prevalence of comorbid depression, suicidality, and substance use.^60,61,62^ Such findings highlight the need for research on the association between AUD and suicide mortality among this specific population, a population not covered in the present study.

### Limitations

This study has a few limitations that should be acknowledged. Titles and abstracts of most of the records were screened independently by a single reviewer, which is a deviation from the PRISMA guidelines. However, this took place following the screeners being trained using batches of 100 randomly selected records, which involved independent review and discussion of all discrepancies, until high agreement (eg, κ > 0.8)^23^ was reached. Further, the measure of association is based on all available data, with no restriction on study year. Given the sociocultural aspects of both AUD and suicide, which can change over time, the association could also vary over time to a certain degree. On a related note, the diagnostic criteria for AUD have changed over the years, which was not possible to control for in the present study. However, as per the forest plots, the association between AUD and suicide mortality does not appear to have varied over time. Also, as would be expected when pooling estimates across populations and settings, we observed high heterogeneity (as per the *I*^2^ statistic) in the meta-analyses. However, it should be noted that the *I*^2^ statistic can be overestimated when summarizing studies with large sample sizes,^63,64^ which is a characteristic of most studies included herein. The likelihood of the *I*^2^ statistic being overestimated is further supported by the small τ^2^ values (a measure of heterogeneity that remains stable regardless of total sample size), which indicate low between-study heterogeneity in all meta-analyses. Last, at the time of publication, the systematic literature search was over a year old. However, the impact on the overall findings is believed to be limited, as the association between AUD and suicide mortality does not appear to have changed notably over time, and the number of eligible studies published within the past year is likely small.

That said, when sufficient time has passed, the present analyses should be updated. Additionally, further stratification by study design should be explored in the future when additional studies become available, as it was not possible to do so in the present study due to the number of studies available (particularly for longitudinal case-control studies). Under the random-effects model, Jackson and Turner^65^ proposed a general rule of thumb that at least 5 studies are required for a meta-analysis that is more informative than the largest individual study of that meta-analysis.

## Conclusions

To our knowledge, this study represents the most up-to-date, comprehensive systematic review and meta-analysis on the sex-specific association between AUD and suicide mortality and is the first to also investigate key methodological moderators (ie, comparator group and study design) of the respective association. Alcohol use disorder is associated with higher odds of death by suicide, an association that is similar across the sexes. It is evident that identifying and treating AUD is an important component of a comprehensive suicide prevention strategy.

## Notes

Supplement 1. **eMethods.** Search Terms Used in PubMed **eTable 1.** Joanna Briggs Institute Critical Appraisal Results for Case-Control Studies **eTable 2.** Joanna Briggs Institute Critical Appraisal Results for Cross-Sectional Studies **eTable 3.** Random-Effects Meta-Regression Model Results **eTable 4.** E-value Estimates for Each Random-Effects Meta-Analysis **eFigure 1.** Risk of Bias in Nonrandomized Studies of Exposure (ROBINS-E) Results (Longitudinal Cohort Studies) **eFigure 2.** Forest Plot of the Effect of AUD on Suicide Mortality Risk Among Males **eFigure 3.** Forest Plot of the Effect of AUD on Suicide Mortality Risk Among Females **eFigure 4.** Funnel Plot for Male Estimates **eFigure 5.** Funnel Plot for Female Estimates

Supplement 2.
Data Sharing Statement
