# Supplementary material for: Sex-Specific Association of Alcohol Use Disorder With Suicide Mortality: A Systematic Review and Meta-Analysis
Source: JAMA Netw Open. 2024 Mar 12;7(3):e241941. doi: 10.1001/jamanetworkopen.2024.1941 (PMC10933726; doi:10.1001/jamanetworkopen.2024.1941)
Supplement: Supplement 1. — eMethods. Search Terms Used in PubMed eTable 1. Joanna Briggs Institute Critical Appraisal Results for Case-Control Studies eTable 2. Joanna Briggs Institute Critical Appraisal Results for Cross-Sectional Studies eTable 3. Random-Effects Meta-Regression Model Results eTable 4. E-value Estimates for Each Random-Effects Meta-Analysis eFigure 1. Risk of Bias in Nonrandomized Studies of Exposure (ROBINS-E) Results (Longitudinal Cohort Studies) eFigure 2. Forest Plot of the Effect of AUD on Suicide Mortality Risk Among Males eFigure 3. Forest Plot of the Effect of AUD on Suicide Mortality Risk Among Females eFigure 4. Funnel Plot for Male Estimates eFigure 5. Funnel Plot for Female Estimates [file jamanetwopen-e241941-s001.pdf]

Supplementary Online Content

Lange S, Kim KW, Lassere AM, et al. Sex-specific association of alcohol use disorder with suicide mortality: a systematic review and meta-analysis. *JAMA Netw Open*. 2024;7(3):e241941. doi:10.1001/jamanetworkopen.2024.1941

eMethods. Search Terms Used in PubMed
eTable 1. Joanna Briggs Institute Critical Appraisal Results for Case-Control Studies
eTable 2. Joanna Briggs Institute Critical Appraisal Results for Cross-Sectional Studies
eTable 3. Random-Effects Meta-Regression Model Results
eTable 4. E-value Estimates for Each Random-Effects Meta-Analysis
eFigure 1. Risk of Bias in Nonrandomized Studies of Exposure (ROBINS-E) Results (Longitudinal Cohort Studies)
eFigure 2. Forest Plot of the Effect of AUD on Suicide Mortality Risk Among Males
eFigure 3. Forest Plot of the Effect of AUD on Suicide Mortality Risk Among Females
eFigure 4. Funnel Plot for Male Estimates
eFigure 5. Funnel Plot for Female Estimates

This supplementary material has been provided by the authors to give readers additional information about their work.

eMethods. Search Terms Used in PubMed

("suicide"[MeSH Terms] OR ("self-harm"[Title/Abstract] OR "suicid*"[Title/Abstract] OR "self injur*"[Title/Abstract])) AND ("alcohol drink*"[Title/Abstract] OR "alcohol intoxication"[Title/Abstract] OR "alcohol abuse"[Title/Abstract] OR "alcohol dependen*"[Title/Abstract] OR "alcohol related disorder*"[Title/Abstract] OR "heavy episodic drink*"[Title/Abstract] OR "alcohol use"[Title/Abstract] OR "alcohol addiction"[Title/Abstract] OR "alcohol consum*"[Title/Abstract] OR "binge drink*"[Title/Abstract] OR "heavy drink*"[Title/Abstract] OR "alcoholi*"[Title/Abstract] OR "alcohol misuse"[Title/Abstract] OR "alcohol abstinen*"[Title/Abstract] OR "alcohol intake"[Title/Abstract] OR "blood alcohol"[Title/Abstract] OR "drunkenness*"[Title/Abstract] OR "blood alcohol content"[MeSH Terms] OR "alcoholic intoxication"[MeSH Terms] OR "alcoholism"[MeSH Terms] OR "drinking behavior"[MeSH Terms])

© 2024 Lange S et al. *JAMA Network Open*.

eTable 1. Joanna Briggs Institute Critical Appraisal Results for Case-Control Studies

Study	Q1	Q2	Q3	Q4	Q5	Q6	Q7	Q8	Q9	Q10
Allebeck, 1987	Yes	Yes	Yes	Yes	Yes	No	No	Yes	Yes	Yes
Dalca, 2013	Yes	Yes	No	Yes	Yes	No	No	Yes	Yes	Yes
Dumais, 2005	Yes	Yes	Yes	Yes	Yes	Yes	Yes	Yes	Yes	Yes
Heu, 2018	Yes	Yes	Yes	Yes	Yes	No	No	Yes	Yes	Yes
Kim, 2003	Yes	Yes	Yes	Yes	Yes	No	No	Yes	Yes	Yes
Kolves, 2006	Yes	Yes	Yes	Yes	Yes	Yes	Yes	Yes	Yes	Yes
Lynch, 2020	Yes	Yes	Yes	Yes	Yes	Yes	Yes	Yes	Yes	Yes
Park, 2008	Yes	Yes	Yes	Yes	Yes	No	No	Yes	Yes	Yes
Penttinen, 2001	Yes	Yes	Yes	Yes	Yes	No	No	Yes	Yes	Yes
Schneider, 2005	Yes	Yes	Yes	Yes	Yes	Yes	Yes	Yes	Yes	Yes
Shaffer, 1996	Yes	Yes	Yes	Yes	Yes	Yes	Yes	Yes	Yes	Yes
Waern, 2003	Yes	Yes	Yes	Yes	Yes	Yes	Yes	Yes	Yes	Yes

Q1. Were the groups comparable other than the presence of disease in cases or the absence of disease in controls?
Q2. Were cases and controls matched appropriately?
Q3. Were the same criteria used for identification of cases and controls?
Q4. Was exposure measured in a standard, valid and reliable way?
Q5. Was exposure measured in the same way for cases and controls?
Q6. Were confounding factors identified?
Q7. Were strategies to deal with confounding factors stated?
Q8. Were outcomes assessed in a standard, valid and reliable way for cases and controls?
Q9. Was the exposure period of interest long enough to be meaningful?
Q10. Was appropriate statistical analysis used?

© 2024 Lange S et al. *JAMA Network Open*.

eTable 2. Joanna Briggs Institute Critical Appraisal Results for Cross-Sectional Studies

Study	Q1	Q2	Q3	Q4	Q5	Q6	Q7	Q8
Yoon, 2011	Yes	Yes	Yes	Yes	No	No	Yes	Yes

Q1. Were the criteria for inclusion in the sample clearly defined?
Q2. Were the study subjects and the setting described in detail?
Q3. Was the exposure measured in a valid and reliable way?
Q4. Were objective, standard criteria used for measurement of the condition?
Q5. Were confounding factors identified?
Q6. Were strategies to deal with confounding factors stated?
Q7. Were the outcomes measured in a valid and reliable way?
Q8. Was appropriate statistical analysis used?

© 2024 Lange S et al. *JAMA Network Open*.

eTable 3. Random-Effects Meta-Regression Model Results

**Male model (k=23)**				
		**Estimate (logOR)**	**95% CI**	***p*-value**
	Intercept	0.88	0.53-1.23	<0.001
Non-exposed group[a]	No AUD (previous or assessed at baseline)	-0.08	-0.65-0.50	0.80
Study design type[b]	Cross-sectional case-control study	0.68	0.08-1.28	0.03
	Prevalence study	-0.55	-1.55-0.46	0.29
	Longitudinal case-control study	0.76	-0.05-1.58	0.07
**Female model (k=17)**				
		**Estimate (logOR)**	**95% CI**	***p*-value**
	Intercept	0.86	0.44-1.27	<0.001
Non-exposed group[a]	No AUD (previous or assessed at baseline)	-0.36	-1.16-0.44	0.38
Study design type[b]	Cross-sectional case-control study	1.41	0.57-2.24	<0.001
	Prevalence study	1.07	0.08-2.05	0.03
	Longitudinal case-control study	0.60	-0.38-1.59	0.23

AUD: Alcohol use disorder

[a]ref= No AUD (lifetime), [b]ref= longitudinal cohort study design

© 2024 Lange S et al. *JAMA Network Open*.

eTable 4. E-value Estimates for Each Random-Effects Meta-Analysis

Model	E-value	Lower bound*
Males, longitudinal studies	4.80	3.12
Females, longitudinal studies	4.21	2.37
Males, cross-sectional studies	6.80	4.38
Females, cross-sectional studies	13.24	12.54

*Upper bound not estimated, as the E-value and lower bound represent the minimum effect size required to shift the pooled estimate to the null

© 2024 Lange S et al. *JAMA Network Open*.

eFigure 1. Risk of Bias in Nonrandomized Studies of Exposure (ROBINS-E) Results (Longitudinal Cohort Studies)

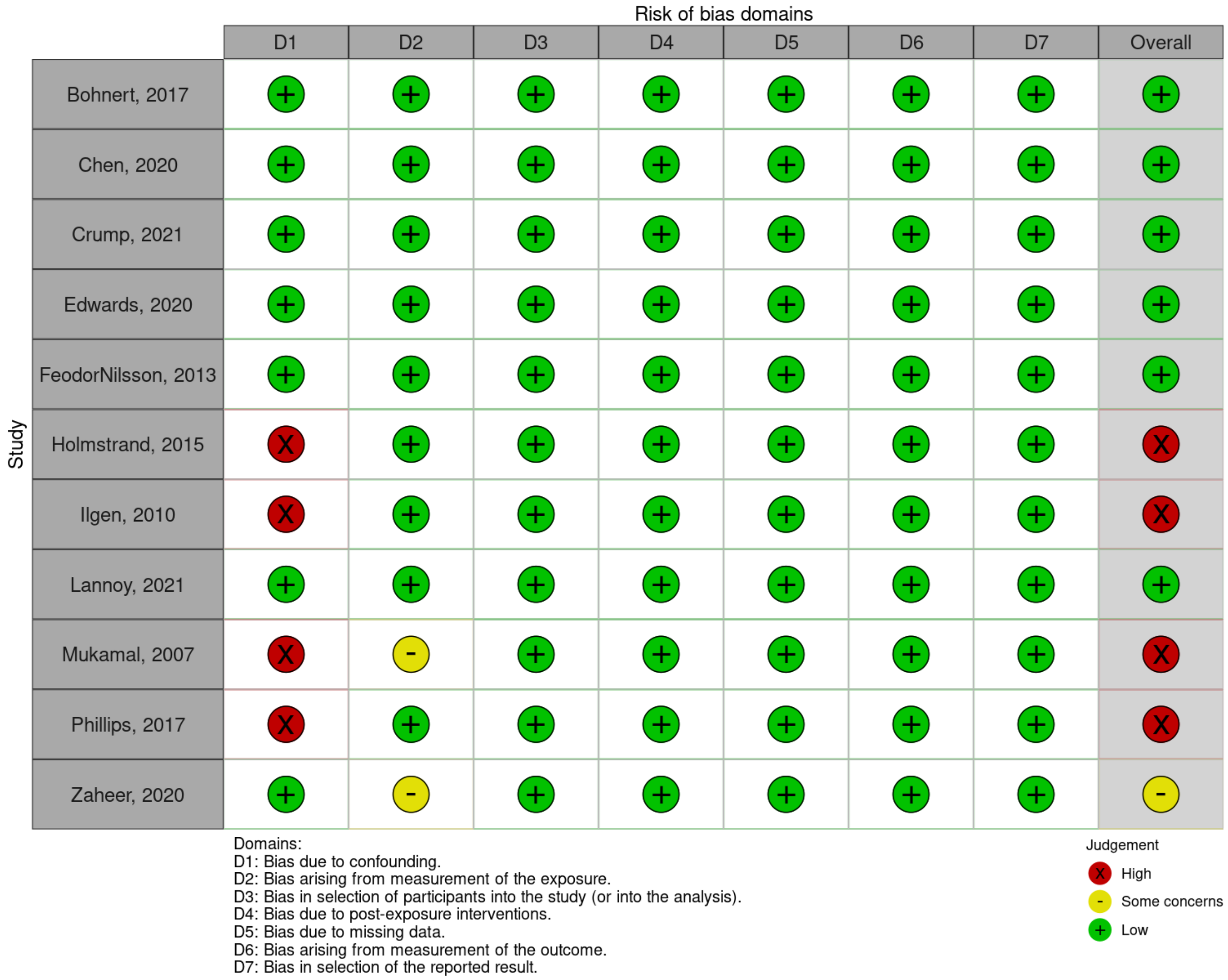

© 2024 Lange S et al. *JAMA Network Open*.

eFigure 2. Forest Plot of the Effect of AUD on Suicide Mortality Risk Among Males

Study	logOR	SE(logOR)	OR	95%−CI	Weight
Allebeck 1987	0.9163	0.7885	2.50	[0.53; 11.73]	2.1%
Shaffer 1996	1.7647	0.5322	5.84	[2.06; 16.57]	3.2%
Penttinen 2001	2.5802	1.1391	13.20	[1.42; 123.07]	1.2%
Kim 2003	1.6503	0.4263	5.21	[2.26; 12.01]	3.8%
Waern 2003	2.1041	1.0651	8.20	[1.02; 66.13]	1.3%
Dumais 2005	1.4061	0.5530	4.08	[1.38; 12.06]	3.1%
Schneider 2005	1.3507	0.3305	3.86	[2.02; 7.38]	4.5%
Kolves 2006	1.9705	0.2309	7.17	[4.56; 11.28]	5.1%
Mukamal 2007	0.8372	0.7618	2.31	[0.52; 10.28]	2.1%
Park 2008	1.9461	0.0921	7.00	[5.85; 8.39]	5.8%
Ilgen 2010	0.8242	0.0369	2.28	[2.12; 2.45]	5.9%
Yoon 2011	0.3334	0.0148	1.40	[1.36; 1.44]	5.9%
Dalca 2013	0.6575	0.3523	1.93	[0.97; 3.85]	4.3%
FeodorNilsson 2013	0.5306	0.1768	1.70	[1.20; 2.40]	5.4%
Holmstrand 2015	1.3376	0.2872	3.81	[2.17; 6.69]	4.8%
Bohnert 2017	0.4947	0.0374	1.64	[1.52; 1.76]	5.9%
Phillips 2017	0.8671	0.3108	2.38	[1.29; 4.38]	4.6%
Heu 2018	0.4318	0.9432	1.54	[0.24; 9.78]	1.6%
Edwards 2020	0.7031	0.0578	2.02	[1.80; 2.26]	5.9%
Lynch 2020	1.5261	0.1057	4.60	[3.74; 5.66]	5.8%
Zaheer 2020	−0.0726	0.1034	0.93	[0.76; 1.14]	5.8%
Crump 2021	0.9042	0.0444	2.47	[2.26; 2.69]	5.9%
Lannoy 2021	2.2246	0.0344	9.25	[8.65; 9.90]	5.9%
**Random effects model**			**3.03**	**[2.31; 3.99]**	**100.0%**

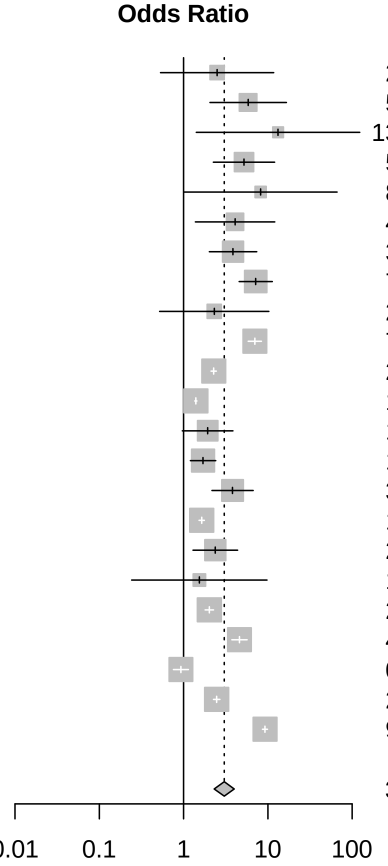

Heterogeneity: $I^2 = 99\%$, $\tau^2 = 0.3280$, $p = 0$

© 2024 Lange S et al. *JAMA Network Open*.

eFigure 3. Forest Plot of the Effect of AUD on Suicide Mortality Risk Among Females

Study	logOR	SE(logOR)	OR	95%−CI	Weight
Allebeck 1987	−0.5108	0.8920	0.60	[0.10; 3.45]	3.5%
Waern 2003	2.5878	1.2312	13.30	[1.19; 148.54]	2.2%
Schneider 2005	1.5810	0.5238	4.86	[1.74; 13.57]	5.8%
Kolves 2006	2.2693	1.0836	9.67	[1.16; 80.89]	2.7%
Park 2008	2.1998	0.3169	9.02	[4.85; 16.79]	7.5%
Ilgen 2010	1.7984	0.1929	6.04	[4.14; 8.82]	8.4%
Yoon 2011	1.9249	0.0267	6.85	[6.50; 7.22]	9.0%
Dalca 2013	1.6487	0.8198	5.20	[1.04; 25.93]	3.8%
FeodorNilsson 2013	0.2624	0.3641	1.30	[0.64; 2.65]	7.1%
Holmstrand 2015	1.0750	1.0432	2.93	[0.38; 22.64]	2.8%
Bohnert 2017	0.4511	0.2261	1.57	[1.01; 2.45]	8.2%
Heu 2018	0.7129	1.2388	2.04	[0.18; 23.12]	2.2%
Chen 2020	0.5596	1.0551	1.75	[0.22; 13.84]	2.8%
Edwards 2020	0.7080	0.0799	2.03	[1.74; 2.37]	8.9%
Lynch 2020	2.3702	0.2692	10.70	[6.31; 18.14]	7.9%
Zaheer 2020	0.0296	0.1768	1.03	[0.73; 1.46]	8.5%
Crump 2021	1.2754	0.0790	3.58	[3.07; 4.18]	8.9%
**Random effects model**			**3.41**	**[2.25; 5.15]**	**100.0%**

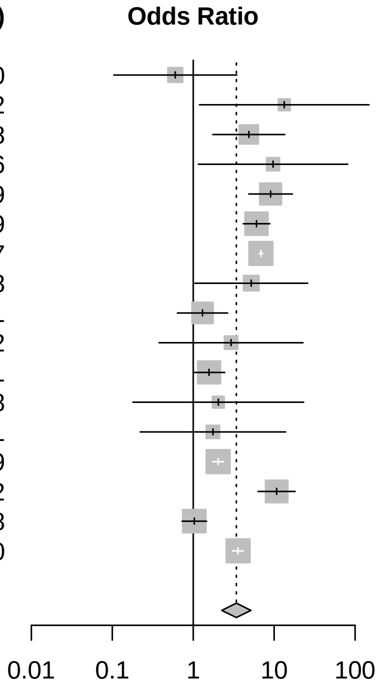

Heterogeneity: $I^2 = 96\%$, $\tau^2 = 0.4945$, $p < 0.01$

© 2024 Lange S et al. *JAMA Network Open*.

eFigure 4. Funnel Plot for Male Estimates

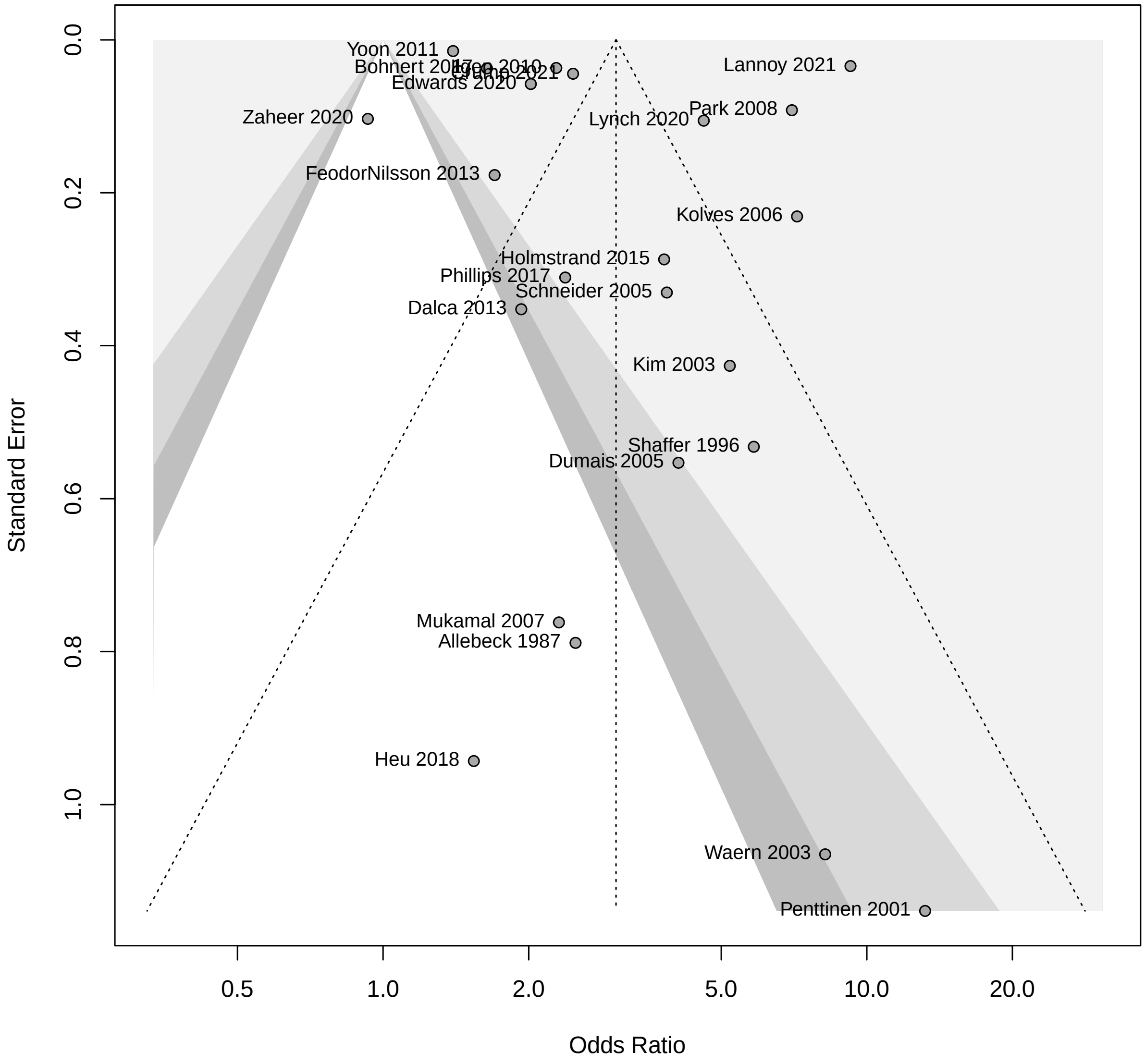

© 2024 Lange S et al. *JAMA Network Open*.

eFigure 5. Funnel Plot for Female Estimates

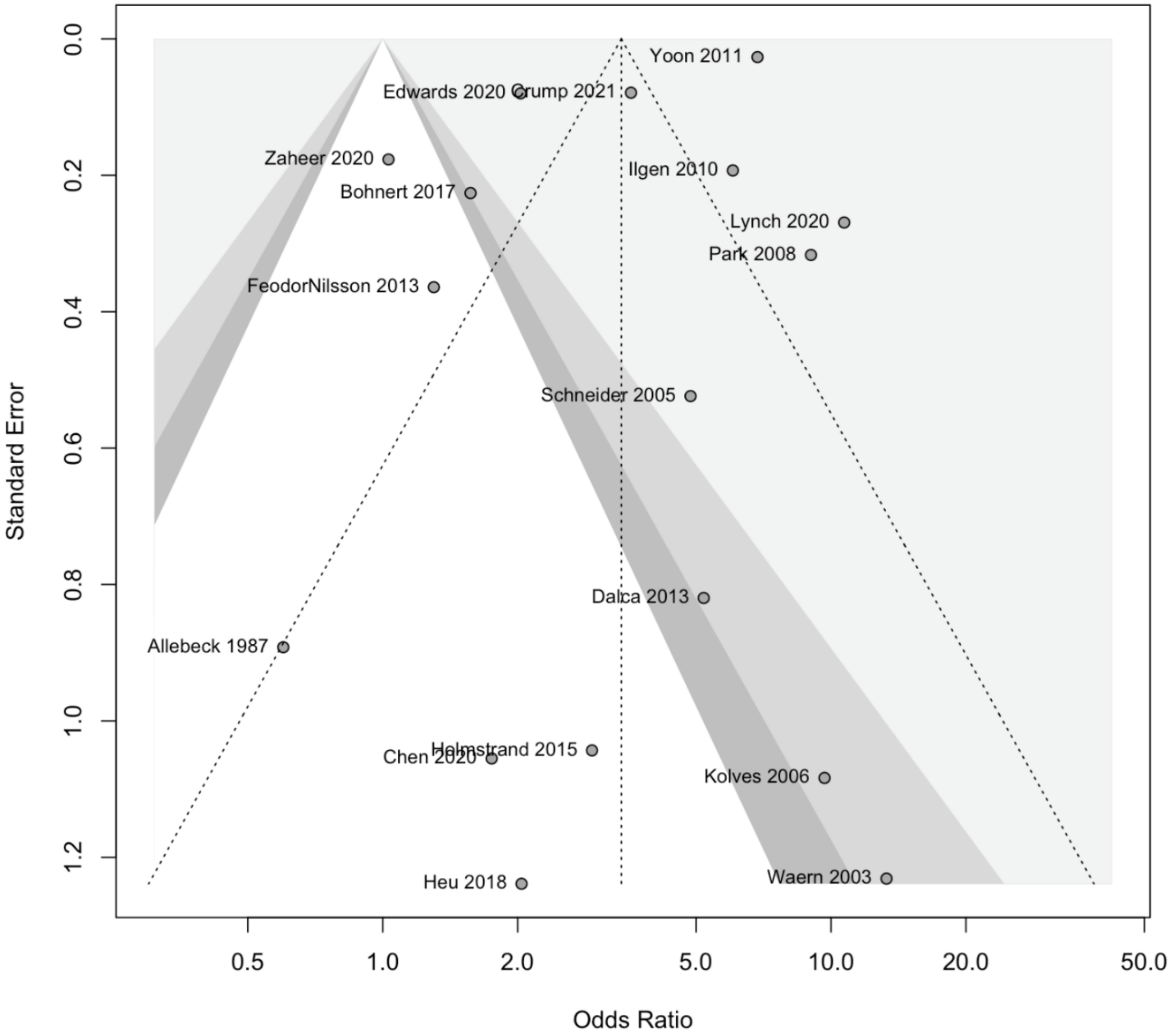

© 2024 Lange S et al. *JAMA Network Open*.
