# Supplementary material for: Sex-Specific Association of Alcohol Use Disorder With Suicide Mortality: A Systematic Review and Meta-Analysis
Source: JAMA Netw Open. 2024 Mar 12;7(3):e241941. doi: 10.1001/jamanetworkopen.2024.1941 (PMC10933726; doi:10.1001/jamanetworkopen.2024.1941)
Supplement: Supplement 2. — Data Sharing Statement [file jamanetwopen-e241941-s002.pdf]

Data Sharing Statement

Lange. Sex-Specific Association of Alcohol Use Disorder With Suicide Mortality. *JAMA Netw Open*. Published March 12, 2024. doi:10.1001/jamanetworkopen.2024.1941

Data
Data available: Yes
Data types: Data (not involving human participants)
How to access data: All data necessary to replicate the findings are included in the Article or Supplementary Material.
When available: With publication

Supporting Documents
Document types: Statistical/analytic code
How to access documents: The R code used to analyse these data can be requested from the corresponding author.
When available: With publication

Additional Information
Who can access the data: Anyone requesting the data.
Types of analyses: For any purpose.
Mechanisms of data availability: Without investigator support.
